# *Morinda citrifolia* L.: A Comprehensive Review on Phytochemistry, Pharmacological Effects, and Antioxidant Potential

**DOI:** 10.3390/antiox14030295

**Published:** 2025-02-28

**Authors:** Silu Hou, Danyang Ma, Shaofeng Wu, Qiaoyue Hui, Zhihui Hao

**Affiliations:** 1 Technology Innovation Center for Food Safety Surveillance and Detection (Hainan), Sanya Institute of China Agricultural University, Sanya 572025, China; 15735151341@163.com (S.H.); danyangma0330@163.com (D.M.); fengws0918@gmail.com (S.W.); qiaoyuehui1@gmail.com (Q.H.); 2 National Key Laboratory of Veterinary Public Health Security, College of Veterinary Medicine, China Agricultural University, Beijing 100193, China; 3 Key Biology Laboratory of Chinese Veterinary Medicine, Ministry of Agriculture and Rural Affairs, Beijing 100193, China

**Keywords:** *Morinda citrifolia* L., antioxidant activity, oxidative stress, chemical composition, extraction method

## Abstract

*Morinda citrifolia* L. (*M. citrifolia*), commonly referred to as noni, a Polynesian medicinal plant with over 2000 years of traditional use, has garnered global interest for its rich repertoire of antioxidant phytochemicals, including flavonoids (kaempferol, rutin), iridoids (aucubin, asperulosidic acid, deacetylasperulosidic acid, asperuloside), polysaccharides (nonioside A), and coumarins (scopoletin). This comprehensive review synthesizes recent advances (2018–2023) on noni’s bioactive constituents, pharmacological properties, and molecular mechanisms, with a focus on its antioxidant potential. Systematic analyses reveal that noni-derived compounds exhibit potent free radical scavenging capacity (e.g., 2,2-Diphenyl-1-picrylhydrazyl/2,2′-azino-bis(3-ethylbenzothiazoline-6-sulfonicacid) (DPPH/ABTS) inhibition), upregulate endogenous antioxidant enzymes (Superoxide Dismutase (SOD), Catalase (CAT), Glutathione Peroxidase (GPx)), and modulate key pathways such as Nuclear factor erythroid 2-related factor 2/Kelch-like ECH-associated protein 1 (Nrf2/Keap1) and Nuclear Factor kappa-B (NF-κB). Notably, polysaccharides and iridoids demonstrate dual antioxidant and anti-inflammatory effects via gut microbiota regulation. This highlights the plant’s potential for innovation in the medical and pharmaceutical fields. However, it is also recognized that further research is needed to clarify its mechanisms of action and ensure its safety for widespread application. We emphasize the need for mechanistic studies to bridge traditional knowledge with modern applications, particularly in developing antioxidant-rich nutraceuticals and sustainable livestock feed additives. This review underscores noni’s role as a multi-target antioxidant agent and provides a roadmap for future research to optimize its health benefits.

## 1. Introduction

In the realm of natural resources, plants have long been a source of remarkable substances with diverse applications. *Morinda citrifolia* L. (*M. citrifolia*), well-known as noni, belongs to the Morinda genus, Rubiaceae family. The scientific plant names were provided by “the Plants of the World Online database” (POWO, https://powo.science.kew.org/ (accessed on 26 June 2024)) and “The World Flora Online” (https://www.worldfloraonline.org/ (accessed on 26 June 2024)). *M. citrifolia* is a green shrub or small tree naturalized in tropical climates, distributed along the southeast coast of China, Southeast Asia, Australia, the Pacific Ocean, and the Caribbean [1].

The fruit of *M. citrifolia* is a traditional Polynesian remedy frequently employed to alleviate symptoms of scabies, abscesses, constipation, diarrhea, and inflammation stemming from diverse etiologies [2]. The rhizome of *M. citrifolia* showcases medicinal properties that are quite similar to those of the traditional Chinese herb *Morinda officinalis*. Emerging evidence indicates that *M. citrifolia* possesses anti-inflammatory, antioxidant, anticancer, and antibacterial properties [2,3,4,5,6]. Anthraquinones, flavonoids, coumarins, polysaccharides, and iridoids have been extracted from various parts of *M. citrifolia*, such as the fruits, roots, and stems exhibiting diverse pharmacological effects [7,8,9,10,11,12]. Juice products have been commercially available in the U.S. market since 1996. In modern medicine, *M. citrifolia* has been utilized to treat various conditions, including diabetes, cardiovascular disease, inflammatory disorders, cancer, obesity, pain relief, and malaria [13,14,15,16,17,18,19]. Multiple studies have shown that *Morinda citrifolia* has antioxidant activity at the same level as vitamin C [20].

The scientific exploration of *Morinda citrifolia* (noni) has garnered significant global research attention, as evidenced by bibliometric analyses. According to Web of Science data, China leads global research output with 129 peer-reviewed publications (as of July 2024); the United States has also provided 127 documents, contributing substantial empirical evidence on its phytochemistry and bioactivity. Emerging research economies, including India (66 publications), Brazil (57 publications), and Thailand (35 publications), have also made notable contributions, particularly in pharmacological validation and food science technology. This robust body of multinational research conclusively positions noni as a plant of exceptional therapeutic and agricultural promise, meriting prioritization as a species for multidisciplinary investigation—spanning ethnopharmacology, metabolic engineering, and sustainable cultivation strategies. The geographical diversity of research efforts underscores its adaptability to tropical ecosystems and its potential to address region-specific health and economic challenges.

In the context of livestock farming, oxidative stress is equally a major challenge. Challenges from environmental, technological, nutritional, and biological stressors lead to excessive production of reactive oxygen species in livestock, which in turn affects their growth, reproduction, and overall health. Exploring the antioxidant potential of natural products like noni in animal husbandry has become an emerging and promising area of research, given the growing demand for sustainable and healthy livestock production. With its proven antioxidant activity, noni may offer new solutions to enhance livestock health and performance by effectively reducing oxidative stress.

This literature review seeks to inform the development and utilization of noni fruit in livestock farming by consolidating existing knowledge on its nutritional and bioactive composition and therapeutic potential, facilitating further in-depth investigations into its mechanism of action. This paper reviews the botanical characterization, traditional uses, phytochemical constituents, compositional analyses, pharmacological properties, and safety studies to provide detailed insights into the biologically active compounds present in *M. citrifolia* based on research published between 2018 and 2023.

## 2. Methodological

**Figure 1:**
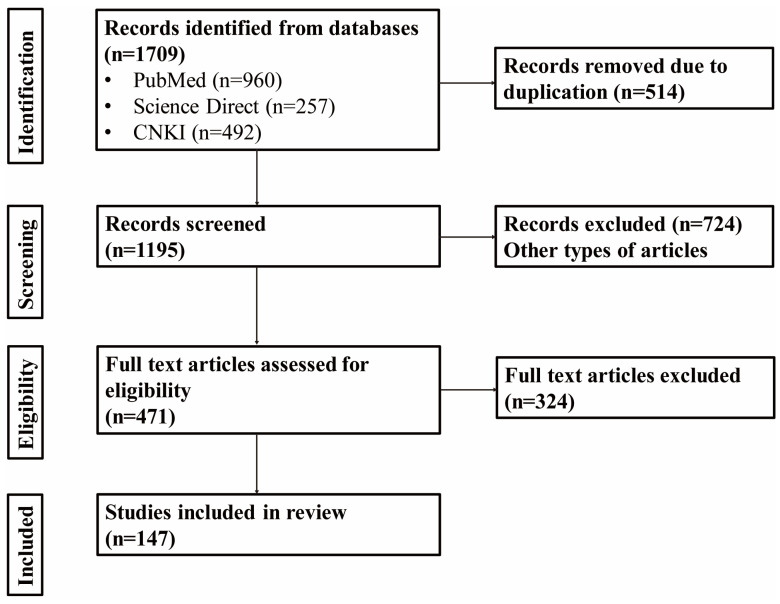
Demonstrating the steps of choosing appropriate articles.

This review comprehensively examines all published articles from the past five years about botanical characterization, traditional uses, phytochemical composition, pharmacological properties, and toxicological studies of *M. citrifolia* in PubMed (https://pubmed.ncbi.nlm.nih.gov/ (accessed on 17 June 2024)), Science-Direct (https://www.sciencedirect.com/ (accessed on 18 June 2024)), and China National Knowledge Infrastructure (CNKI) (https://www.cnki.net/ (accessed on 17 June 2024)). The search terms such as “*Morinda citrifolia* L.”, “Noni”, “Phytochemistry”, “Pharmacology”, and “safety” were employed, and articles were screened and evaluated for relevance based on their titles, abstracts, and keywords. With the application of preferred reporting items for systematic reviews and meta-analysis (PRISMA) guidelines (Figure 1), a total of 147 articles were screened for this review. These articles come from various countries, with 68 articles of noni studied in China, 33 from the U.S., 30 from Brazil, 23 from India, 17 from Cuba, 17 from Indonesia, 16 from Malaysia, 16 from Thailand, and 10 from South Korea. They will be used to provide an in-depth analysis of the phytochemical composition, ethnomedicinal value, and phytopharmacological activity of *M. citrifolia*, offering a comprehensive overview of the current research landscape.

## 3. Botanical Features

**Figure 2:**
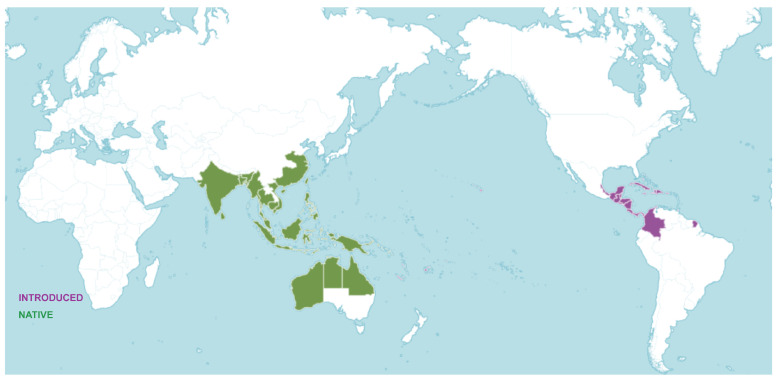
Global distribution of *M. citrifolia* (Source: https://powo.science.kew.org/taxon/urn:lsid:ipni.org:names:756359-1 (accessed on 20 June 2024)).

*M. citrifolia* belongs to the Rubiaceae family, known as “*Hai Ba Ji Tian*” in China. *M. citrifolia* is native to Southeast Asia, widely distributed in the west to India and Sri Lanka, east to Polynesia, south to northern Australia, and north to Jiangsu, China. It is also distributed in the Caribbean, Mexico, and South America. China’s main producing areas are Yunnan, Hainan, and Taiwan. *M. citrifolia* is an evergreen tropical plant, fruiting throughout the year [21]. *M. citrifolia* is a perennial shrub or small arbor with a height of up to 1–5 m and straight stems and branches similar to the shape of a quadrangular column. It can adapt to harsh weather and soil conditions, even at altitudes up to 215 m [1]. *M. citrifolia* is widely distributed because of its light seeds and specialized structure, which enables it to spread by wind or water currents [22]. The global geographic distribution of *M. citrifolia* is shown in Figure 2.

The fruit of *M. citrifolia* (noni) is a multiple fruit consisting of fused drupes, each containing four seeds, which develop between 9 months and 1 year after the tree is planted [23]. Noni is known as “nuna” in India [21], “mengkudu” in Malaysia [24], and “cheese fruit” in Australia [25]. Noni is oval in shape and consists of many small fruits clustered together, which measure approximately 5 to 10 cm in length and 3 to 6 cm in width. Each fruit may contain up to 260 seeds [1]. Initially, the fruits are green when young, and the skin is hard and rough in the early stage of maturity, turning white when ripe and finally turning yellowish–white and softening after ripening [21]. When the fruits are in the late stage of ripening, they have a rather unpleasant odor. The *M. citrifolia* bears fruit throughout the year, although the yield decreases in winter. The leaves of *M. citrifolia* are abundant and dark green in color, with rounded, spindly blades ranging from 8 to 25 cm in length [26]. Its flowers are white, tubular, smaller, clustered, and borne on peduncles.

## 4. Phytochemistry

The diverse chemical composition of medicinal plants is the key to their pharmacological activity. Studies on the phytochemistry of *M. citrifolia* have focused on the roots, leaves, and fruits, with anthraquinones, iridoids, flavonoids, and coumarins as the main compounds; among these, damnacanthal, scopoletin, rutin, ursolic acid, and asperuloside are the main components of *M. citrifolia* [26,27,28]. The compounds isolated from the roots are dominated by anthraquinone compounds [29] (e.g., damnacanthal [12] and sterols [30]). The *M. citrifolia* leaves contain a variety of iridoids, flavonoids, and triterpenoids [9,31,32,33]. Noni juice as a dietary supplement is the reason why more focus has been placed on the compositional analysis of the fruit, including phenols, polysaccharides, coumarins, fatty acids, cyclic enol ether terpenes, flavonoids, carotenoids, essential oils, and more [10,11,34], and the composition of vitamins, amino acids, and trace minerals has also been reported [1]. Table 1 summarizes the information on the chemical composition of *M. citrifolia* from different studies.

### 4.1. Iridoids

Iridoids are a fascinating group of compounds that can be found in many different types of plants and insects, such as the Aspidistra, Gentianae, Lamiaceae, Lauraceae, Rubiaceae, Sedum, and Verbenaceae families, as well as in insects, including butterflies [68]. Iridoids are a subclass of monoterpenoids and are acetal derivatives of iridodial. Natural iridoids possess the basic skeleton of a cyclopentadiene-fused pyran ring. Iridoids can be divided into iridoid glycosides, secoiridoids glycosides, bis-iridoids, and non-glycosidic iridoids. Iridoids have been shown to have many different biological activities, including anti-inflammatory, antibacterial, antiviral, neuroprotective, hepatoprotective, hypoglycemic, antitumor, and antioxidant activities [69,70].

**Figure 3:**
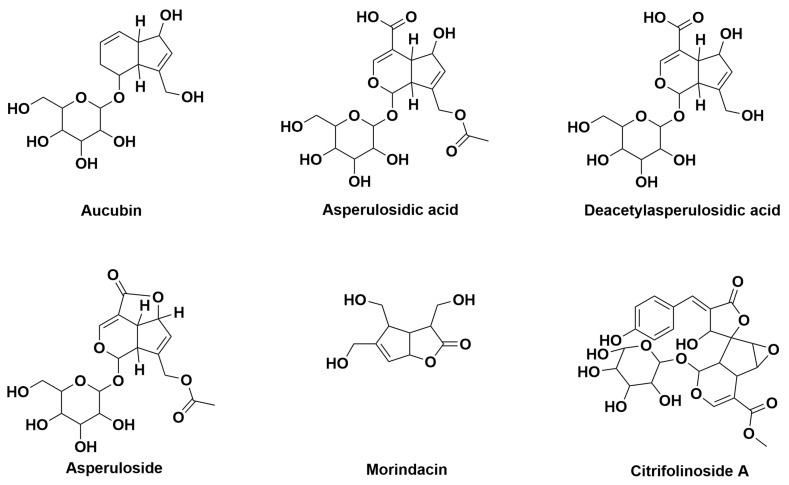
Structures of iridoids from *M. citrifolia*.

Figure 3 shows the six main types of iridoids contained in *M. citrifolia*: aucubin, asperulosidic acid, deacetylasperulosidic acid, asperuloside, morindacin, citrifolinoside A.

### 4.2. Anthraquinones

Anthraquinones are a class of chemical compounds that consist of three benzene rings fused together in a specific arrangement. They are widely found in nature and can be found in various plants such as Polygonum and Rubiaceae, particularly in the rhizomes and bark of plants, as well as in certain fungi [71]. In *M. citrifolia*, anthraquinones are one of its primary bioactive substances [72]. They are primarily present in the roots of *M. citrifolia*.

The presence of hydroxyl groups on the anthraquinone ring is associated with a range of biological activities, including anti-tumor, antimicrobial, antioxidant, and anti-osteoporosis effects. The polarity of the substituent groups on the anthraquinone ring is directly related to its antimicrobial activity; generally, the stronger the polarity of the substituent, the more effective the antimicrobial ability. However, the substitution of sugar chains on anthraquinones tends to reduce their antioxidant activity [73].

**Figure 4:**
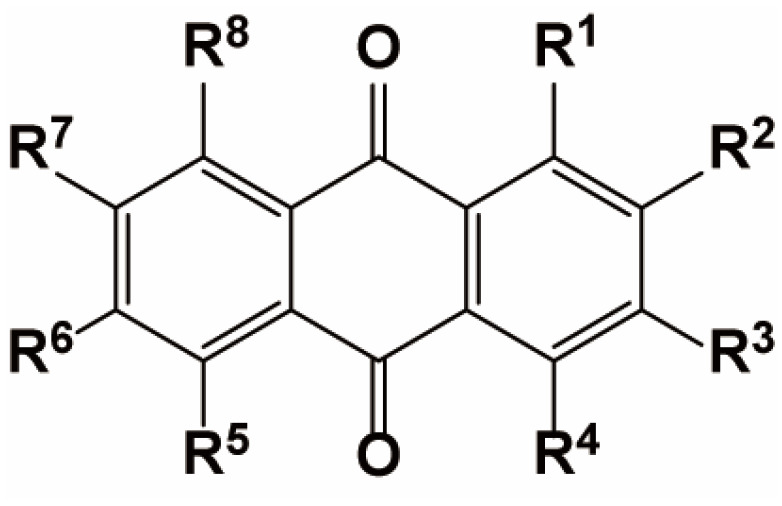
Structures of anthraquinones from *M. citrifolia*.

The most significant anthraquinone found in *M. citrifolia* is damnacanthal. Its ability to target various tyrosine kinases shows promising anti-cancer activity [74]. Other anthraquinones isolated from *M. citrifolia* include alizarin, morindadiol, nordamnacanthal, rubiadin, ibericin, tectoquinone, lucidin, damnacanthol-*ω*-ethyl ether, lucidin-*ω*-butyl ether, rubiadin-dimethyl ether, rubiadin-1-methyl ether, rubiadin-3-methyl ether, and 1-hydroxy-2-methyl-9,10-anthraquinone. For more information on the detailed chemical structure formula of these compounds, please refer to Figure 4 and Table 2.

### 4.3. Coumarins

**Figure 5:**
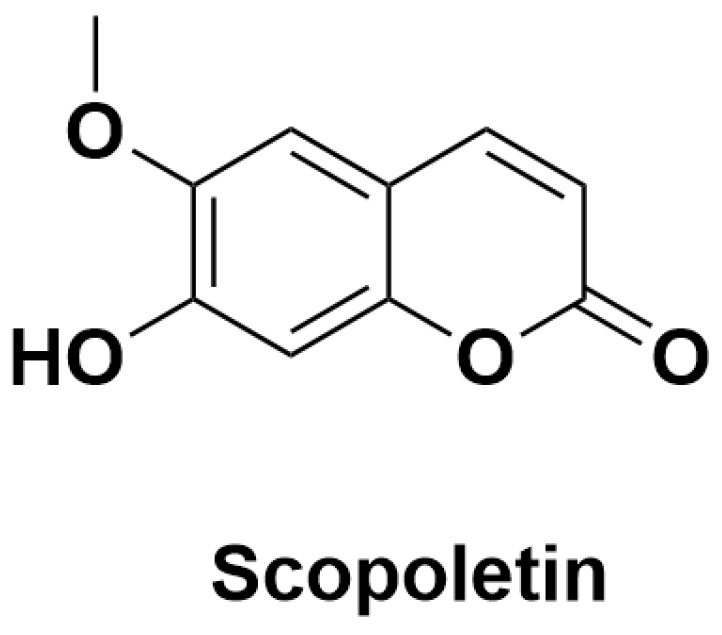
Structures of scopoletin from *M. citrifolia*.

Coumarins possess a unique bicyclic structure consisting of a benzene ring and a pyran ring. Variations in the substituents on the bicyclic ring contribute to a diverse range of biological properties and activities, including antioxidant, antibacterial, and anti-inflammatory effects. Coumarins are commonly found in plants from the Leguminosae, Rosaceae, and Cuscuta families [75]. For instance, scopoletin is the primary coumarin present in *M. citrifolia*, and there is 6 mg/g in the water extract of its leaves [76]. The structural formula of scopoletin is shown in Figure 5.

### 4.4. Flavonoids

Flavonoids are plant-specific secondary metabolites, which are small-molecule natural products with a variety of pharmacological effects such as anticancer, antioxidant, anti-inflammatory, and reducing vascular fragility. The basic structure of flavonoids is two aromatic rings and a three-carbon bridge connected to a diphenylpropane structure with phenolic or polyphenolic groups in various positions. Assessing the quality of noni extract involves considering its total flavonoid content [14], which is typically measured using rutin standards for assays [77]. Interestingly, the flavonoid content of noni extract can vary slightly depending on the ripeness of the fruit, with a consistent upward trend observed as noni ripens. In a study by Zhang [78] and Chen [77], the total flavonoid content of noni juice and noni fruits was found to be 234.42 mg/L and 12.56–14.48 g/kg.

**Figure 6:**
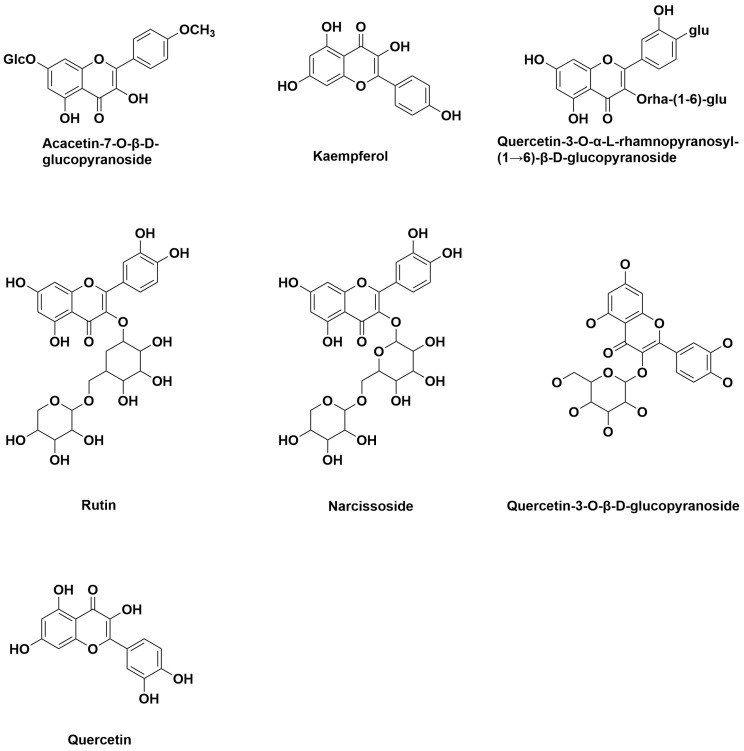
Structures of flavonoids from *M. citrifolia*.

The following flavonoid compounds are found in *M. citrifolia* (Figure 6): acacetin-7-*O*-*β*-D-glucopyranoside, kaempferol, rutin, narcissoside, quercetin, quercetin-3-*O*-*β*-D-glucopyranoside, quercetin-3-*O-α*-L-rhamnopyranosyl-(1→6)-*β*-D-glucopyranoside, and 5,7-dumethyl-apigenin-4′-*O*-*β*-D-galactopyranoside.

### 4.5. Polysaccharide

**Figure 7:**
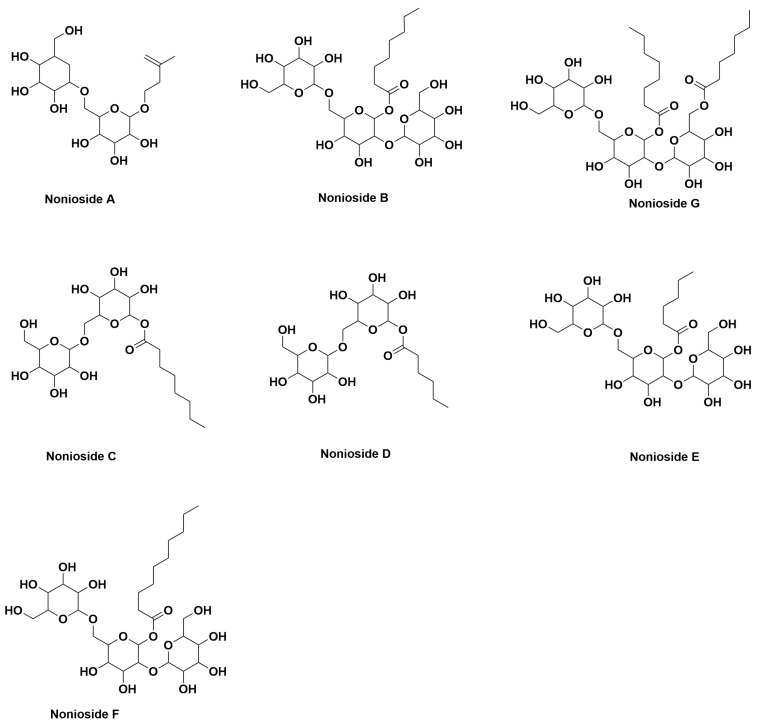
Structures of polysaccharide from *M. citrifolia*.

Polysaccharides are molecules containing more than ten monosaccharides connected by glycosidic bond polymerization and are important biomolecules in nature. They have biological activities such as immunomodulation, antioxidant, anti-inflammatory, anti-tumor, and hypoglycemic activity. Various types of noni extracts have been researched and found to have good anti-inflammatory and immunomodulatory properties because of polysaccharides. Extraction of noni polysaccharides is usually done by extracting the juice or puree with distilled water, centrifugation of the extract, and precipitation with alcohol [20,79,80,81,82]. The crude polysaccharide content in noni fruit decreases significantly as it matures, from 1.72 g/100 g to 1.07 g/100 g. Research suggests that enzymes in noni break down the polysaccharides in the cell wall, resulting in a gradual decrease in polysaccharide content as the fruit softens. Figure 7 shows the structure of polysaccharides isolated from *M. citrifolia* fruits.

### 4.6. Nutrients

Minerals account for approximately 8.4% of the dry matter of noni fruit and can vary depending on the fruit’s maturity. West et al. [35] found that potassium is the most abundant mineral in noni puree, with a content of approximately 214.34 mg/100 g. In addition, other minerals such as calcium, iron, sodium, and selenium have also been detected. Vitamins have been identified in studies related to noni fruit as well. Noni fruit has the highest content of vitamin C, ranging from 24 to 158 mg per 100 g of dry matter [21]. The vitamin C content in noni puree is approximately 1.13 mg/g.

## 5. Extraction Method for *M. citrifolia*

In order to explore the phytochemistry of *M. citrifolia* and conduct pharmacological research, numerous methods have been employed to extract the various compounds present in *M. citrifolia*. These techniques encompass Soxhlet extraction [83], ultrasound-assisted extraction [84], solid-phase microextraction [11], cold-soak extraction [85], subcritical water extraction [86], and microwave-assisted extraction [87]. The choice of solvent plays a pivotal role in the extraction of phytochemicals. The polarity of the solvent is closely related to the solubility of the target ingredient. Fat-soluble ingredients (e.g., anthraquinones) are commonly used, such as *n*-hexane [12], dichloromethane [88], and ethyl acetate. Medium polar components (e.g., flavonoid glycosides) are mostly solvated with methanol [47,55], ethanol [31,56,68] (95%), or a mixture [84] thereof. Water-soluble components (e.g., polysaccharides [29]) are enriched by high-temperature aqueous [89] extraction combined with ethanol precipitation (4 °C), supplemented by large-pore resin decolorization/deproteinization for purification [79,90,91].

Column chromatography, liquid chromatography, and thin-layer chromatography have been utilized to isolate specific compounds [47]. In column chromatography, mobile phases like H_2_O-MeOH, EtOAc-MeOH, *n*-hexane-chloroform, and chloroform-ethyl acetate are commonly employed. Medium-pressure liquid chromatography (MPLC) is also used to extract phytochemicals from dichloromethane extracts of *M. citrifolia*, employing gradient elution with petroleum ether, chloroform, and chloroform enriched with methanol (1%, 2%, and 5%) [88]. Sigma-Aldrich thin-layer chromatography (TLC) plate preparation thin-layer chromatography is utilized to purify bioactive phytochemicals from distinct fractions [29]. Tan [92] utilized a mobile phase of toluene/ethyl acetate/formic acid in the ratio of 5:4:1 to extract compounds like caffeic acid, linalool, kaempferol, and gallic acid in combinations of noni, *Coriandrum sativum* L., and *Aegle marmelos* (L.) Corrêa.

The identification of both quantitative and qualitative aspects of isolated products is conventionally carried out through the use of high-performance liquid chromatography coupled with ultraviolet and mass spectrometry (HPLC-UV/MS) [31] and HPLC-MS. Identification of different compounds is achieved by comparing their HPLC retention time, UV absorbance of target peaks, and mass/charge ratio. Deng [31] extracted noni leaves by diafiltration, and the extracts were analyzed by HPLC. Four flavonoids in the extracts were identified by comparing the retention times and the subsequent characterization of the target peaks by MS and UV spectroscopy against the standards. For compounds of unknown structure, after isolation and purification, structural analysis is usually carried out by nuclear magnetic resonance (NMR) and high-resolution fast atom bombardment- mass spectrometry (HRFAB-MS) spectroscopy. Hu [56] isolated and purified two white powders from the ethanolic extract of noni fruit, and the two glycosides were identified by NMR spectroscopy as *(2E*,*4E*,*7Z)*-deca-2,4,7-trienoate-2-*O*-*β*-D-glucopyranosyl-*β*-D-glucopyra-noside and amyl-1-*O*-*β*-D-apio-furanosyl-1,6-*O*-*β*-D-glucopyranoside, and Wang [68] analyzed three new compounds using 1D and 2D NMR spectra with TMS as an internal standard: methyl 3-(2,4-dihydroxy-5-methoxyphenyl) propionate, butyl 3-(2,4-dihydroxy-5-methoxyphenyl)propionate, and 5-hydroxyhexyl 2-hydroxypropanoate.

The antioxidant efficacy of *M. citrifolia* extracts is critically influenced by the following extraction techniques:

Ultrasound-assisted extraction (UAE) achieves higher yields of polyphenols (e.g., scopoletin) and iridoids (e.g., asperulosidic acid) due to cavitation-induced cell wall disruption, preserving thermo-sensitive antioxidants [11].

Microwave-assisted extraction (MAE) excels in polysaccharide recovery (∼85% efficiency) through rapid dielectric heating yet may degrade heat-labile flavonoids [80].

A recent comparative study demonstrated that UAE extracts exhibited superior DPPH radical scavenging activity (IC_50_: 12.3 μg/mL) compared to MAE (IC_50_: 18.7 μg/mL), highlighting the need for method-specific optimization based on target compounds [20].

## 6. Traditional Use

**Figure 8:**
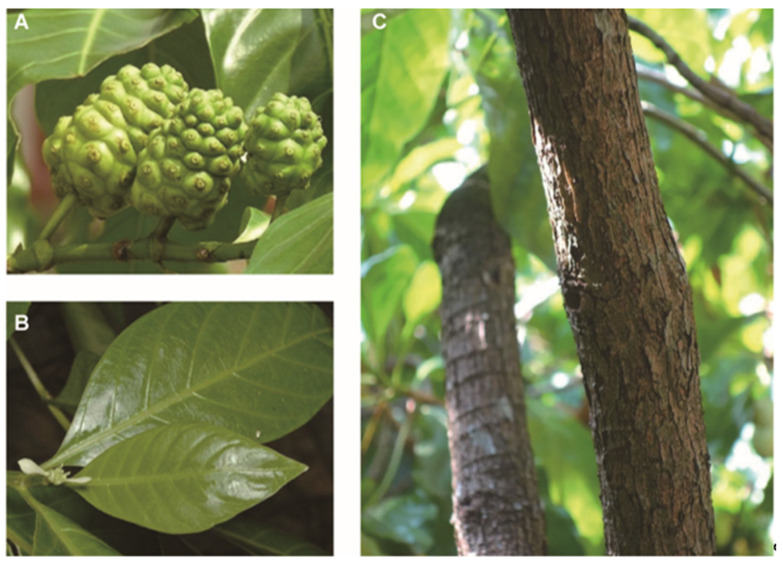
The green fruits (**A**), leaves (**B**), and bark (**C**) of *M. citrifolia*.

The Tahitians discovered the medicinal value of *M. citrifolia* around 400 A.D. Traditional remedies mainly utilize the bark, leaves, and green fruits primarily for localized disease treatment [2]. Figure 8 shows images of different parts of the *M. citrifolia* plant. The bark of *M. citrifolia* is used for bacterial infections or abortions. *M. citrifolia* leaves are employed in addressing bacterial infections, inflammation, abdominal pain, and diarrhea [93]. Green fruits are utilized for various purposes, including combating bad breath, bacterial or fungal infections, menstrual cramps, arthritis, stomach ulcers, mouth ulcers, toothache, and indigestion. Ripe fruits can treat parasitic or bacterial infections and serve as an emmenagogue or for bowel cleansing. In the Republic of Palau, noni is frequently utilized as a traditional remedy for diabetes and hypertension. These remedies are typically available in the form of decoctions, juices, or can be chewed directly [94]. Pikad Tri-phol-sa-mut-than is an ancient traditional herbal formula [95], also written as Phikud Tri-Phon (PTP), a combination of noni, coriander, and dried mukul fruits in a 1:1:1 ratio (*w:w*), which can be used as a tonic for treating gastrointestinal ailments, fevers, and trichotomies as well as an antiemetic or laxative.

Despite their unpleasant taste and bitter flavor, noni serves as a viable option during periods of crop failure and food scarcity. Locals on certain Pacific islands consume fresh or cooked noni as a staple food [96]. Additionally, indigenous populations in Southeast Asia and Australia enjoy the raw fruits with salt or incorporate them into curry dishes. The seeds of noni are edible as well, but they should be toasted over a fire before consumption. Rich in vitamins, noni has been used as a health tonic or medicinal beverage by South Pacific islanders for over two thousand years, making it a valuable tropical fruit resource [26].

## 7. Pharmacological Activity of *M. citrifolia*

**Figure 9:**
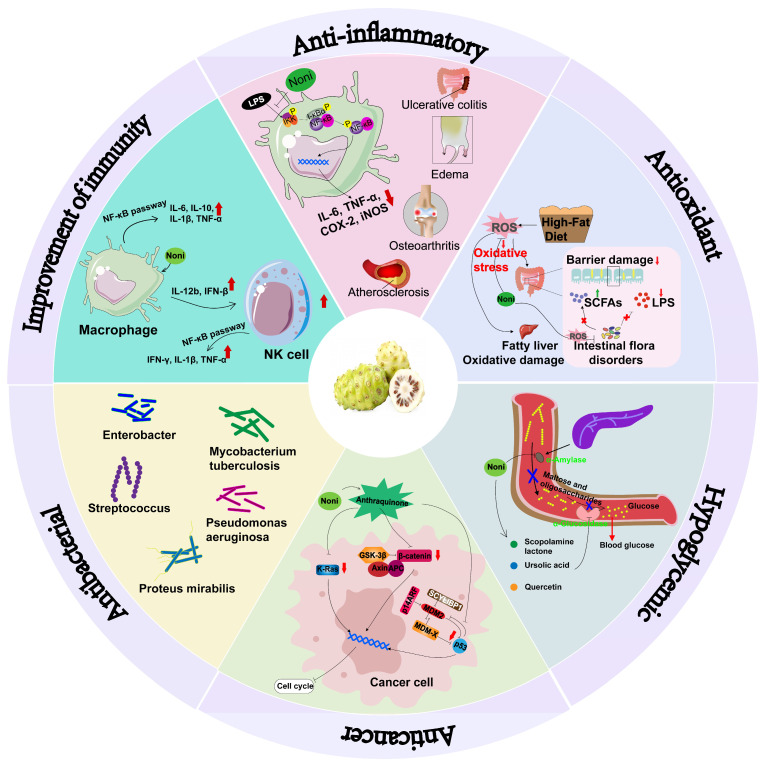
Pharmacological activities of *M. citrifolia*. Upward-pointing arrows denote upregulated expression levels of cytokines/proteins or elevated damage indicators, whereas downward-pointing arrows represent corresponding downregulation of these biological parameters.

A systematic analysis of the existing literature reveals that current investigations on *Morinda citrifolia* predominantly focus on its multifaceted pharmacological activities, including anti-inflammatory, antioxidant, anticancer, and antimicrobial effects. These properties are mechanistically linked to its rich repertoire of bioactive compounds, such as anthraquinones, flavonoids, and polysaccharides. As summarized in Figure 9, the pharmacological profile of *M. citrifolia* encompasses both in vitro and in vivo evidence, highlighting its therapeutic potential across diverse disease models.

### 7.1. Antioxidant Activity

Reactive oxygen species (ROS) are produced under physiological conditions as a byproduct of mitochondrial energy production and as an important “weapon” in phagocytosis. The antioxidant defense network is responsible for maintaining low basal levels of ROS by scavenging and converting them into nontoxic products and other mechanisms. Oxidative stress, triggered by the accumulation of excess ROS, is a well-documented contributor to various inflammatory and necrotic processes that are pivotal in the development of numerous diseases and injuries. To assess the antioxidant potential of compounds, DPPH and ABTS scavenging, as well as the measurement of antioxidant enzyme expression (like SOD and GPx), analyses are conducted in vitro. Animal models of high-fat diet-induced oxidative damage in the liver are used in vivo. In the case of *M. citrifolia*, polyphenol, iridoids, scopoletin, and polysaccharide components are recognized as the main antioxidants. Phenolic extract of *M. citrifolia* fruits significantly increases the activities of antioxidant enzymes to effectively attenuate oxidative stress in a high-fat diet-induced nonalcoholic fatty liver disease (NAFLD) mouse, such as glutathione (GSH) and catalase (CAT) [97]. In another study, antioxidant properties of silver nanoparticles synthesized from Gerbera leaf extract were observed in vitro [98]. Additionally, the DPPH free radical scavenging capacity of the Thai traditional group formula PTP (consisting of *A. marmelos*, *M. citrifolia*, and *C. sativum*) was assayed with an IC_50_ of 92.4 ± 6.6 μg/mL [99]. Noni wine was found to regulate the Nrf2 pathway and increase the expression of antioxidant enzyme genes in high-fat diet mice [78].

Notably, the molecular weight of noni polysaccharides has a significant impact on their antioxidant efficacy [100]. Recent studies demonstrate that noni polysaccharides (NFP) exert potent antioxidant effects by scavenging ROS (e.g., DPPH radical inhibition rate > 70%) and activating the Nrf2/Keap1 pathway. In high-fat diet models, NFP significantly upregulated hepatic SOD and CAT levels while reducing malondialdehyde (MDA), suggesting its dual role in mitigating oxidative stress and inflammation via gut microbiota modulation [79]. The preservation of noni through fermentation is an ancient technique that not only reduces its unpleasant odor but also enhances its immune-boosting and antioxidant properties. Zhou [20], Li [80], and Zhang [101] found that noni polysaccharides and their derivatives have DPPH radical neutralization rates over 70% in vitro. This scavenging capacity highlights the potential of noni polysaccharides as potent antioxidants capable of reducing oxidative stress in biological systems.

In one study [102], the ethanolic extract of *M. citrifolia* leaves treated liver damage rats by elevating liver antioxidant enzymes. Further research investigated the antioxidant activity of lignin present in *M. citrifolia* leaves; the findings suggest that lignin is highly effective in capturing free radicals because of phenolic groups [103]. A summary of antioxidant activity studies for *M. citrifolia* can be found in Table 3.

### 7.2. Anti-Inflammatory Activity of M. citrifolia

Inflammation is a crucial factor in the development of many chronic and severe diseases. Available research suggests that *M. citrifolia* exerts its anti-inflammatory effects through multiple pathways (Table 4) via its active components, including phenolic compounds, polysaccharides, and specific proteins. The anti-inflammatory effects of *M. citrifolia* have been validated in multidimensional experimental models such as those for lipopolysaccharide-induced cellular models, inflammatory bowel disease, mouse esophagitis models, rheumatoid arthritis, pneumonia in mice, edema models, and steatohepatitis.

In an in vitro cellular model, noni seed extract reduced tumor necrosis factor-α (TNF-α) and inducible nitric oxide synthase (iNOS) in lipopolysaccharide (LPS)-stimulated RAW 264.7 cells [108]. Furthermore, PTP, a traditional Thai herbal formulation containing *M. citrifolia*, demonstrated notable inhibitory effects on various inflammatory models. PTP extract exhibited significant inhibitory effects on ethyl phenylpropionate-induced rat ear edema and carrageenan or arachidonic acid-induced rat hind paw edema [92]. In another study, fermented noni juice reduced the expression of NLR family pyrin domain-containing protein 3 (NLRP3) and TNF-α and attenuated ankle edema caused by monosodium urate, demonstrating its therapeutic activity in acute gouty arthritis [109]. Phenolic compounds extracted from ripe fruits can reduce the level of intestinal inflammatory factors caused by a high-fat diet [110]. This finding corroborates the research conducted by Dyah Aninta Kustiarini [111]. *M. citrifolia* polysaccharide can inhibit the NF-κB signaling pathway and regulate the intestinal flora, which reduces intestinal damage and treats inflammatory bowel disease [91]. Lastly, deacetylasperulosidic acid (DAA), a cyclic enol ether terpene compound abundant in *M. citrifolia* fruits, concentration-dependently controlled mitogen-activated protein kinase (MAPK) phosphorylation in TNF-α and interferon-γ (IFN-γ)-treated human adult cutaneous T-cell lymphoma (HaCaT) cells during in vitro studies, suggesting that DAA is a potential anti-atopic dermatitis candidate for restoring T helper cell 1/T helper cell 2 (Th1/Th2) immune balance and enhancing skin barrier function [112].

The stems and seeds of *M. citrifolia*, the non-medicinal parts, had anti-inflammation activity due to bioactive compounds. It has been found that noni stem bark effectively iNOS, thereby inhibiting nitric oxide (NO) production and exerting anti-inflammatory activity [113]. Research has revealed that noni seeds, typically discarded in the juice production process, exhibit significant anti-inflammatory capabilities. Further investigation [18] identified a heat-stable anti-inflammatory lipid transfer protein, *M. citrifolia* lipid transfer protein (McLTP_1_), present in noni seeds, which was confirmed to mitigate the side effects associated with irinotecan-induced intestinal mucositis [114].

**Table 4:** Summary of studies on anti-inflammatory activity of *M. citrifolia*.

No.	Parts	Animal/Cell	Model	Application Part or Compounds	Dose	Pharmacological Activity	Reference
1	Seeds	RAW 264.7 cells	LPS-induced inflammation	Cannabidiol, *M. citrifolia* seed extract	100 μg/mL	Reduction of the expression of inflammatory mediators	[113]
2	Fruits	C57BL/6 mice	High-fat diet-induced obesity	Phenol extract	100, 200 mg/kg	Enhancement of gut microbiome balance and intestinal barrier integrity, coupled with a decrease in inflammatory responses	[110]
3	Fruits	MH7A cells	N/A	Pyranone derivatives, alkaloids, lignans, glycosides	3.69–168.96 µmol/L	Inhibition of MH7A synovial fibroblast proliferation in vitro	[85]
4	Fruits	SD rat	Ethyl phenylpropiolate-induced ear edema, carrageenan- and arachidonic acid-induced hind paw edema	PTP extract	150, 300, 600 mg/kg	Effective reduction of various inflammatory mediators	[115]
5	Seeds	Swiss mice	Irinotecan-stimulated intestinal mucositis	McLTP_1_	8 mg/kg	Lipid transfer protein McLTP_1_ significantly prevents irinotecan-induced intestinal damage, reduces intestinal muscle hypercontractility, and has anti-inflammatory activity	[92]
6	Fruits	Kunming mice	Sodium urate-induced acute gouty arthritis	*M. citrifolia* L. fruit juice	7.8 mL/kg	Modulation of miRNA molecules with the ability to regulate the development of acute gouty arthritis and reduction in inflammatory cytokines interleukin 6 (IL-6), interleukin 8 (IL-8), and TNF-α in acute gouty arthritis in mice	[114]
7	Fruits	C57BL/6 mice	Dextran sulfate sodium induced inflammatory bowel disease	Polysaccharide	10 mg/kg	Noni polysaccharides containing homogalacturonan and rhamnogalacturonan-I structural domains may alleviate inflammatory bowel disease (IBD) by modulating the gut microbiota and inhibiting inflammation-related signaling pathways	[91,109]
8	Stems, Leaves	MH7A cells	N/A	Sesquiterpenes, steroids, lignans, and fatty acids	38.69–203.45 μmol/L	Inhibition of MH7A synovial fibroblast proliferation	[91]
9	Seeds	RAW 264.7 cells	N/A	Cannabidiol, *M. citrifolia* seed extract	100 μg/mL	Reduction in the expression of inflammatory mediators	[116]
10	Fruits	HaCaT, Human Mast Cell-1 (HMC-1) and Eosinophilic Leukemia-1 (EOL-1) Cells	LPS-induced inflammation	Deacetyl asperulosidic acid, asperulosidic acid	0.01, 0.05, 0.1, 0.2 μmol/L	Activation of the NF-κB signaling pathway, regulation of Th1/Th2 immune balance, and enhancement of skin barrier function	[112]
11	Fruits, Leaves, Seeds	RAW 264.7 cells	High-fat diet induced oxidative stress, inflammation, and intestinal dysbiosis in the liver	Noni fruit, leaf extract, noni fruit seed extract, noni fruit essential oil	100 μg/mL	Reduction in expression of nitric oxide synthase (NOS) and pro-inflammatory cytokine TNF-α	[108]
12	Fruits	SD rat	Acetic acid-induced colitis	Polysaccharide	100 mg/kg	Modulation of intestinal flora and SCFA production, reduction of colonic barrier permeability and metabolic endotoxemia, thereby attenuating hepatic oxidative stress and inflammation in HFD rats	[79]
13	Fruits	Swiss mice	LPS-induced inflammation	Polysaccharide	3 mg/kg	Reduction in inflammatory cell infiltration, oxidative stress, pro-inflammatory cytokines, COX-2, and iNOS expression in the inflamed colon	[90]
14	Fruits	RAW 264.7 cells	Intra-articular monosodium iodoacetate injection into the right knee induced osteoarthritis	Asperulosidic acid, rutin, nonioside A, (*2E*,*4E*,*7Z*)-deca-2,4,7-trienoate-2-*O*-*β*-D-glucopyranosyl-*β*-D-glucopyranoside, tricetin	50 µg	Activation of iNOS and COX-2 and regulation of NO production	[117]
15	Leaves	SD rat	Mannitol-induced arthritis	*M. citrifolia* leaves extract	400 mg/kg	Inhibition of inflammation, NO production, protease-induced joint catabolism, and oxidative stress	[118]
16	Fruits	Saccharomyces cerevisiae Kinase Gene (SKG) mice	IBD induced by 2% sucrose solution	Noni fruit juice	Fruit juice mixed with drinking water in equal proportions	Suppression of arthritis and histopathology scores	[111]
17	Fruits	Wild-type Groningen rat	COVID-19	30.56% Colostrum and 11.11% *M. citrifolia* juice blend	0.5 g/kg	Reduction in interleukin-10 (IL-10), interleukin-12 (IL-12), and TNF-α protein	[119]
18	Fruits	Patients	N/A	Papaya and noni fruit fermented syrup	14 g/d	Reduction in IL-6, IL-8, and NO metabolites	[120]
19	Fruits	RAW 264.7 cells, Balb/c mice	N/A	Fermented noni fruit polysaccharide	100, 200 mg/kg	Increasing NO and pro-inflammatory cytokine release, activation of COX-2 and iNOS	[121]
20	Fruits	RAW 264.7 cells, C57BL/6 mice	Prednisolone-induced immunosuppression in rats LPS-induced Tohoku hospital pediatrics-1 (THP-1) macrophages	Noni fruit ethanol extract	50, 200 mg/kg	Activation of NF-κB and Activator Protein-1 (AP-1) pathways and increasing mRNA expression of IL-6, IFN-β, TNF-α, interleukin-1 beta (IL-1β) and IL-12b in RAW 264.7 cells	[122]
21	Fruits	Balb/c mice, The human monocyte cell line (THP-1, ATCC TIB-202)	2,6-Dinitro-1-chlorobenzene (DNCB) induced atopic dermatitis-like lesions	Noni fruit ethanol extract	200 mg/kg	Downregulating LPS-induced TNF-α, IL-1β, and IL-6/IL-10, while attenuating iNOS and NF-κB expression	[123]
22	Fruits	NC/Nga mice	N/A	Fermented Noni Juice	250, 500, 1000 mg/kg	Regulation of the Th1/Th2 immune balance as well as the T helper cell 17 (Th17) and T helper cell 22 (Th22) immune responses and reduction of the infiltration of inflammatory cells	[124]
23	Fruits	RAW264.7 cells, C57BL/6 mice	LPS-induced inflammation	*M. citrifolia* fruit water extract	0–400 μg/mL	Activation of NF-κB and AP-1 signaling pathways, induction of NO, regulation of immune cell populations, induction of pro-inflammatory cytokine gene expression, and inhibition of IL-10 expression	[125]

Notes: N/A, data not available.

### 7.3. Anticancer Activity of M. citrifolia

Over the last five years, hot spots on noni juice and its extracts have included anticancer studies. Utilizing various cancer cell lines and combining computer simulation technology, these studies aim to explore the inhibitory effects of *M. citrifolia* on cancer cell growth and proliferation (Table 5). Compounds in *M. citrifolia*, like anthraquinone, have shown excellent anticancer activity, such as nordamnacanthal, damnacanthal morindone, and rubiadin [126]. In a comprehensive study, researchers identified eight anthraquinone compounds that demonstrated in vitro proliferative suppression comparable to that of adriamycin on various tumor cell lines, HL-60 human promyelocytic leukemia cell line (HL-60), Shanghai Medical College of Fudan University hepatocellular carcinoma-7721 (SMMC-7721), A-549, michigan cancer foundation-7 (MCF-7), and SW480 human colon cancer cell line (SW480), owing to the interaction of these compounds with key residues of multiple targets via carbonyl oxygen or van der Waals forces, which has been elucidated by molecular docking methods [127].

Reports indicate that the anthraquinone compounds extracted from *M. citrifolia* have selective activity against colorectal cancer cells [6]; morindone and rubiadin exhibited binding affinity towards targets like *β*-catenin, murine double minute 2-p53 protein (MDM2-p53), and kirsten rat sarcoma viral oncogene homolog (KRAS). By molecular docking, pharmacophore modeling, induced-fit docking, and molecular dynamics simulations, five compounds with anti-hepatocellular carcinoma activities, namely soranjidiol, thiamine, lucidin, 2-methyl-1,3,5-Trihydroxyanthraquinone, and rubiadin, could be screened out from the compounds of *M. citrifolia* by using B-Raf kinase as a target [128]. A study of lung cancer further found that noni juice inhibits the expression of proliferation proteins such as Ki67 in tumor cells and promotes the expression of apoptosis-related genes to play an anti-lung cancer role [129], the promotion of apoptosis of cancer cells by *M. citrifolia* extract was also observed in leukemic mice [130]. However, further research is needed to fully explore their efficacy against human cancer.

**Table 5:** Summary of studies on anticancer activity of *M. citrifolia*.

No.	Parts	Animal/Cell	Model	Application Part or Compounds	Dose	Pharmacological Activity	Reference
1	Fruits	HL-60, SMMC-7721, A-549, MCF-7, and SW480 tumor cell lines	N/A	New anthraquinone: moricitrifone and 7 other known anthraquinones	0.26–16.58 μmol/L	Antiproliferative activities	[127]
2	Roots	HCT116 human colon cancer cell line (HCT116), LS174T human colon cancer cell line (LS174T), HT29 human colon cancer cell line (HT29)	N/A	Morindone, rubiadin	0.25–64 μM	Cytotoxicity to colorectal cancer cells	[6]
3	Fruits	A-549 cells, BLAB/c nu/nu mice	A-549 cells were inoculated into the right axilla	Noni juice	in vitro: 50 μL/mL; in vivo: 0.2 mL/10 g, 0.4 mL/10 g	AKT/NF-κB signaling pathway	[129]
4	Seeds	A-549 and human prostate cancer (LNCaP) cell lines	N/A	MCEO-CHs NPs (nanoparticles of noni fruit essential oil loaded with chitosan)	5–25 μL/mL	Cytotoxicity to cancer cells	[131]
5	Leaves	In vitro: Jurkat leukemia and Walter and Eliza Hall institute-3B (WEHI-3B) cell lines; in vivo: Balb/c mice	Leukemic	Ethanol extract	in vitro: 1.562–100 μg/mL; in vivo: 100, 200 mg/kg	Affecting immunity, suppressing inflammation, and upregulating apoptosis	[130]

Notes: N/A, data not available.

### 7.4. Hypoglycemic Activity of M. citrifolia

For many years, noni has been used in Indonesia and Polynesia as a natural remedy for diabetes. To investigate this traditional use scientifically, diabetes models are usually induced using a combination of a high-fat, high-sugar diet, streptozotocin (50 mg/kg BW), and tetracycline (Table 6). The study revealed that noni components had therapeutic activity for type 2 diabetes by inhibiting *α*-amylase and *α*-glucosidase activities and exerting insulin-like effects [132]. Human pancreatic *α*-amylase is a crucial target for treating type 2 diabetes mellitus. In this context, utilizing molecular docking and molecular dynamics simulations [133], ursolic acid demonstrated optimal binding energy in molecular docking simulations with *α*-amylase, and molecular dynamics confirmed this, suggesting its potential as a therapeutic agent for diabetes management. Xu [134] identified 15 compounds in noni seeds that showed *α*-glucosidase inhibitory activity. Another study [135] examined the mixtures of noni, mango, and pineapple; the sample was tested for *α*-glucosidase inhibitory activity, as well as *α*-amylase inhibitory activity using acarbose as a control. The results of this study suggested that the combination of these three fruit extracts possessed exceptional in vitro antidiabetic activity and was further confirmed in an in vivo validation assay of the streptozotocin-induced diabetes model in SD rats.

### 7.5. Antimicrobial Activity of M. citrifolia

Antimicrobial activity is usually assessed using the disk diffusion method (Table 7). Dried *M. citrifolia* root powders were sequentially extracted by hexane, dichloromethane, ethyl acetate, and ethanol; the inhibition zone showed that hexane extracts have inhibitory effects on *S. aureus*, *S. epidermidis*, and *B. cereus*, while the dichloromethane extract exhibited the inhibition of *E. coli* with an inhibition zone of 5.5 mm, the ethyl acetate extract of the root showed inhibition against *P. aeruginosa* and *S. epidermidis*. They also tested the antitubercular activity of *M. citrifolia* using a microplate Alamar Blue assay and found that the ethanol extract of *M. citrifolia* leaves showed 89% inhibition of *M. tuberculosis* at a 100 μg/mL concentration [3]. As a dental disinfectant [137], the water extract of *M. citrifolia* was shown to disinfect better than conventional disinfectant and photodynamic therapy in a model of dental metal–ceramic crown disinfection. Because of this, the cerium oxide nanoparticles of noni assembled exhibit bacteriostatic activity against Gram-positive and Gram-negative bacteria and were more effective than amoxicillin [138].

## 8. Safety Studies on *M. citrifolia*

As of July 2024, no prescription or over-the-counter (OTC) drug using noni as the main active ingredient has been formally approved by mainstream international drug regulatory agencies (e.g., U.S. Food and Drug Administration (FDA), European Medicines Agency (EMA) of the European Union, China National Medical Products Administration (NMPA)). Currently, the drug-level development of noni-related ingredients is still in the early research stage, and some of the progress includes the following: Animal experiments have shown that noni polysaccharides may improve insulin resistance, but they have not yet entered the anti-diabetic clinical trials. Anthraquinone components (e.g., damnacanthal) have shown in vitro inhibitory effects on hepatocellular carcinoma and colon cancer cells, but clinical data are lacking. Iridoids (e.g., asperuloside) are well-studied for their anti-inflammatory mechanisms but have not entered the drug development pipeline.

Although *M. citrifolia* has been used as a complementary treatment for cancer and is liver protective [141,142,143], the safety of *M. citrifolia* is controversial. Cases of *M. citrifolia* acute hepatitis-like illnesses have been reported [144,145], while in vivo and in vitro studies have shown no evidence of hepatotoxicity or acute hepatocellular injury [146]. The research on genotoxicity found that *M. citrifolia* fruit and seed substances have no mutagenic potential to induce chromosome damage [147]. Similarly, the safety tests on commercial noni juice demonstrated no adverse effects on the liver for both humans and animals. These findings suggest that noni fruits and seeds are not mutagenic or disruptive [148].

Meanwhile, in a reproductive toxicity test [149], it was observed that the administration of high doses of *M. citrifolia* water extracts to pregnant rats resulted in liver damage, along with adverse reproductive effects. These effects included anticonception properties, intrauterine growth restriction, and fetal malformations, highlighting potential risks to both the mother and the fetus. This is contrary to the results that the subcutaneous injection of water extracts of *M. citrifolia* in mice does not cause toxicity to the reproductive system [150]. This discrepancy between the studies underscores the complexity of the impact of noni on the reproductive system. Given these conflicting results, further toxicological research is warranted. Such studies will be crucial for establishing a comprehensive safety profile and guiding the responsible use of noni extracts.

## 9. Discussion

The primary thrust of *M. citrifolia* research is to unravel the health benefits of noni juice or its extracts, aiming to pinpoint its potential as a remedy for various human diseases [151,152,153]. In diseases, *M. citrifolia* extracts activate NF-κB and AP-1 pathways and down-regulate inflammatory factors (IL-6, IFN-β, TNF-α, and IL-1β) [112].

*M. citrifolia* polysaccharides (NFPs) have emerged as key bioactive compounds that mitigate oxidative stress through dual mechanisms: direct free radical scavenging and indirect modulation of gut microbiota. Studies reveal that NFP promotes the proliferation of beneficial bacteria (e.g., Lactobacillus and Bifidobacterium), which ferment dietary fiber to produce short-chain fatty acids (SCFAs) such as butyrate. Butyrate activates the Nrf2/Keap1 pathway, upregulating antioxidant enzymes (SOD, CAT, GPx) while suppressing ROS generation. Additionally, NFP enhances intestinal barrier integrity by increasing tight junction proteins (e.g., occludin), thereby reducing systemic endotoxin leakage and subsequent oxidative damage [91]. These findings position *M. citrifolia* as a potent modulator of the gut–liver axis in combating oxidative stress-related pathologies.

This review aims to provide a comprehensive overview that bridges the gap between traditional knowledge and scientific understanding, shedding light on the multifaceted applications of *M. citrifolia* across human and animal healthcare. The multifaceted antioxidant properties of *M. citrifolia* underpin its potential in diverse industries, such as the following.

Functional Foods: Incorporation of noni polysaccharides into nutraceuticals could address diet-induced oxidative stress, significantly increasing the antioxidant liver enzymes SOD and GPx [81].

Cosmeceuticals: Topical formulations containing noni seed (50%) ethanolic extract demonstrate anti-aging effects by inhibiting matrix metalloproteinase-1 (MMP-1) and down-regulation of MAPK phosphorylation in UVA-irradiated normal human dermal fibroblasts [154].

Livestock Production: As a feed additive, noni powder (0.2% *w*/*w*) reduced oxidative damage in broilers exposed to heat stress, improving feed conversion ratio and meat quality via lipid peroxidation inhibition [155]. Challenges such as odor palatability may be overcome through microencapsulation or fermentation-based masking techniques. It can improve livestock physiology and performance [156]. Adding noni fruit powder to the diet could promote the growth of black goats and improve immunity and antioxidant ability [157] because noni fruit polysaccharides may help ruminants metabolize nutrients [158] and alleviate rumen acidosis [159]. The addition of noni has also been reported in broiler and Holstein cow diets [155,160]. *M. citrifolia* is an extremely valuable natural source of antioxidants. Studies have found that coccidian infections can cause oxidative stress due to the production of ROS; certain phytopharmaceuticals with anticoccidial properties can provide some protection against this stress. For instance, the acetone extract of the traditional Chinese medicine *Morinda officinalis* has been shown to reduce oxidative damage and effectively prevent and control avian coccidiosis [161]. Noni and *Morinda officinalis* are closely related as they both belong to the genus Morinda. *M. citrifolia* contains phenolic compounds, saponins, essential oils (including terpenes and their derivatives) [162], and other compounds that have demonstrated anticoccidial effects through various mechanisms of action.

## 10. Conclusions

Future research should prioritize three key areas. Firstly, it is crucial to gain mechanistic depth by elucidating how noni-derived compounds synergistically regulate the crosstalk between oxidative stress and inflammation. Secondly, application-driven studies are needed to develop nanoformulations or fermented products to enhance the bioavailability and stability of antioxidant constituents. Thirdly, sustainable utilization should be emphasized, specifically by validating noni’s role as a livestock feed additive to mitigate oxidative damage in agriculture, which aligns with One Health initiatives. By integrating traditional wisdom with cutting-edge science, *M. citrifolia* holds immense potential as a sustainable, multi-target antioxidant agent, offering novel strategies for combating oxidative stress-related diseases and promoting global health.

## Figures and Tables

**Table 1:** Compounds isolated from *M. citrifolia*.

Class	No.	Compounds	Molecular Formula	Parts	References
Aminoacids	1	Alanine	C_3_H_7_NO_2_	Fruits	[35]
	2	Arginine	C_6_H_14_N_4_O_2_	Fruits	
	3	Aspartic acid	C_4_H_7_NO_4_	Fruits	
	4	Cysteine	C_3_H_7_NO_2_S	Fruits	
	5	Glutamic acid	C_5_H_9_NO_4_	Fruits	
	6	Glycine	C_2_H_5_NO_2_	Fruits	
	7	Histidine	C_6_H_9_N_3_O_2_	Fruits	
	8	Isoleucine	C_6_H_13_NO_2_	Fruits	
	9	Leucine	C_6_H_13_NO_2_	Fruits	
	10	Lysine	C_6_H_14_N_2_O_2_	Fruits	
	11	Methionine	C_5_H_11_NO_2_S	Fruits	
	12	Phenylalanine	C_9_H_11_NO_2_	Fruits	
	13	Proline	C_5_H_9_NO_2_	Fruits	
	14	Serine	C_3_H_7_NO_3_	Fruits	
	15	Threonine	C_4_H_9_NO_3_	Fruits	
	16	Tryptophan	C_11_H_12_N_2_O_2_	Fruits	
	17	Tyrosine	C_9_H_11_NO_3_	Fruits	
	18	Valine	C_9_H_11_NO_2_	Fruits	
Anthraquinones	19	1,2-Dihydroxy-3-methoxy-anthraquinone	C_15_H_10_O_5_	Roots	[1]
	21	1,3,6-Trihydroxy-2-methylanthraquinone	C_15_H_10_O_4_	Roots	[36]
	22	1,3-Dihydroxy-2-methoxy-anthraquinone	C_15_H_10_O_5_	Roots	[37]
	23	1,3-Dihydroxy-5-methoxy-2,6-bismethoxymethyl-9,10-anthraquinone	C_19_H_18_O_7_	Barks	[38]
	24	1,3-Dihydroxy-5-methoxy-6-methoxymethyl-2-methyl-9,10-anthraquinone	C_18_H_16_O_6_	Barks	
	25	1,3-Dimethoxy-2-methoxymethylanthraquinone	C_18_H_16_O_5_	Roots	[29]
	26	1,3-Dimethoxyanthraquinone	C_16_H_12_O_4_	Fruits	[39]
	27	1,4-Dimethoxyl-2-hydroxyanthraquinone	C_16_H_12_O_5_	Barks	[38]
	28	1,5,15-Trimethylmorindol	C_18_H_16_O_6_	Barks, Fruits, Leaves	
	30	1,5,7-Trihydroxy-6-methoxy-2-methoxymethylanthraquinone	C_17_H_14_O_7_	Fruits	[8]
	31	1,8-Dihydroxy-2-hydroxymethyl-5-methoxyanthraquinone	C_16_H_12_O_6_	Fruits	[28]
	32	1,8-Dihydroxy-2-methyl-3,7-dimethoxyanthraquinone	C_17_H_14_O_6_	Plants	[40]
	33	1-Hydroxy-2-methyl-9,10-anthraquinone	C_15_H_10_O_3_	Roots, Fruits	[1,37]
	35	1-Hydroxy-2-methylol-anthraquinone	C_15_H_10_O_3_	Roots	[39]
	36	1-Hydroxy-2-primeverosyloxymethyl-anthraquinone-3-olate	C_26_H_27_O_14_	Roots	[41]
	37	1-Hydroxy-5,6-dimethoxy-2-methyl-7-primeverosyloxyanthraquinone	C_28_H_32_O_15_	Roots	
	38	1-Hydroxy-5-methoxyanthraquinone	C_15_H_10_O_4_	Barks	[38]
	39	1-Methoxy-2′,2′-dimethyldioxine-(5,6′:2,3)-anthraquinone	N/A	Roots	[1]
	40	1-Methoxy-2-primeverosyloxymethyl-anthraquinone-3-olate	C_27_H_29_O_14_	Roots	[41]
	41	1-Methoxy-3-hydroxyanthraquinone	C_15_H_10_O_4_	Roots	[39]
	42	1-Methyl-3-hydroxy-anthraquinone	C_15_H_10_O_3_	Roots	
	43	1-*O*-gentiobiose-2-methylol-anthraquinone	C_27_H_30_O_14_	Roots	[37]
	44	1-*O*-gentiobiose-3-hydroxy-2-methyl-anthraquinone	C_27_H_30_O_13_	Roots	
	45	1-*O*-gentiobiose-8-methoxy-aloeemodin	C_28_H_32_O_15_	Roots	
	46	1-*O*-gentiobiose-emodin	C_27_H_30_O_15_	Roots	
	47	1-*O*-primeverose-2-methyl-3,6,8-trihydroxy-anthraquinone	C_26_H_28_O_15_	Roots	
	48	1-*O*-primeverose-2-methylol-3-hydroxy-8-methoxy-anthraquinone	C_27_H_30_O_14_	Roots	
	49	1-*O*-primeverose-2-methylol-anthraquinone	C_26_H_28_O_13_	Roots	
	50	1-*O*-primeverose-3,8-dihydroxy-2-methyl-anthraquinone	C_26_H_28_O_14_	Roots	
	51	1-*O*-primeverose-3,8-dimethoxy-2-methyl-anthraquinone	C_28_H_32_O_14_	Roots	
	52	1-*O*-primeverose-3-methoxy-8-hydroxy-2-methylol-anthraquinone	C_27_H_30_O_15_	Roots	
	53	1-*O*-primeverose-8-hydroxy-ibericin	C_28_H_32_O_15_	Roots	
	54	1-*O*-primeverose-aloeemodin	C_26_H_28_O_14_	Roots	
	55	1-*O*-primeverose-emodin	C_26_H_28_O_14_	Roots	
	56	1-*O*-primeverose-rubiadin	C_26_H_28_O_13_	Roots	
	57	1-*O*-*β*-D-glycopyranosyl-8-methoxy-emodin	C_22_H_22_O_10_	Roots	
	58	1-*O*-*β*-D-glycopyranosyl-emodin	C_21_H_20_O_10_	Roots	
	59	1-*O*-*β*-D-glycopyranosylrubiadin-3-methyl ether	C_22_H_22_O_9_	Roots	
	60	2-Ethoxy-1-hydroxyanthraquinone	C_16_H_12_O_9_	Roots	[39]
	61	2-Formyl-1-hydroxyanthraquinone	C_15_H_8_O_4_	Roots	
	62	2-Formylanthraquinone	C_15_H_8_O_3_	Roots	
	63	2-Methoxy-1,3,6-trihydroxyanthraquinone	C_15_H_10_O_6_	Roots	[28]
	64	2-Methoxy-3-methyl-anthraquinone	C_16_H_12_O_3_	Roots	[38]
	65	2-Methyl-4-hydroxy-5,7-dimethoxyanthraquinon	N/A	Flowers	[14]
	66	3-Hydroxy-2-hydroxymethyl- anthraquinone	N/A	Roots	[39]
	67	3-*O*-gentiobiose-1-hydroxy-2-methyl-anthraquinone	C_27_H_30_O_14_	Roots	[37]
	68	3-*O*-primeverose-1,6,8-trihydroxy-2-methyl-anthraquinone	C_26_H_28_O_15_	Roots	
	69	5,15-Di-*O*-methylmorindol	C_17_H_14_O_6_	Fruits	[39]
	70	6-Hydroxy-anthragallol-1,3-dimethylether	C_16_H_12_O_6_	Fruits	
	71	8-*O*-gentiobiose-emodin	C_27_H_30_O_15_	Roots	[37]
	72	Alizarin	C_14_H_8_O_4_	Fruits, Roots	[42]
	73	Alizarin-1-methyl ether	C_15_H_10_O_4_	Fruits, Roots	[39]
	74	Aloe-emodin	C_15_H_10_O_5_	Roots	[37]
	75	Anthragallol-2-methyl ether	C_15_H_10_O_5_	Fruits	[39]
	76	Damnacanthal	C_16_H_10_O_5_	Leaves, Roots	[29,42]
	77	Damnacanthol-11-*O*-*β*-primeveroside	N/A	Roots	[41]
	78	Damnacanthol-3-*O*-*β*-D-primeveroside	N/A	Roots	[43]
	79	Damnacanthol-*ω*-ethyl ether	N/A	Roots	[44]
	80	Degiferruginol-11-*O*-*β*-Primeveroside	N/A	Roots	[41]
	81	Digiferruginol-1-Methylether-11-*O*-*β*-gentiobioside	N/A	Roots	
	82	Emodin	C_15_H_10_O_5_	Roots	[37]
	83	Emodin 1-*O*-*β*-D-glycopyranosyl	C_21_H_20_O_10_	Roots	
	84	Fridamycin E	C_19_H_16_O_7_	Roots	
	85	Ibericin	C_17_H_14_O_5_	Roots	[29]
	86	Lucidin	C_15_H_10_O_5_	Fruits	[1]
	87	Lucidin 1,3-dimethyl ether	N/A	Barks	[38]
	88	Lucidin 3-methyl ether	C_16_H_11_O_5_	Barks	
	89	Lucidin-3-*O*-*β*-D-Primeveroside	N/A	Roots	[43]
	90	Lucidin-*ω*-butyl ether	N/A	Roots	[44]
	91	Morindadiol	N/A	Fruits	[45]
	92	Morindicininone	C_17_H_14_O_5_	Stems	[39]
	93	Morindicinone	C_18_H_16_O_6_	Stems	
	94	Morindone	C_15_H_10_O_5_	Fruits	
	95	Morindone-5-methylether	C_16_H_12_O_5_	Fruits	
	96	Morindone-6-methyl-ether	C_16_H_12_O_5_	Barks, Roots	[12]
	97	Morindone-6-*O*-*β*-D-primeveroside	N/A	Roots	[43]
	98	Nordamnacanthal	C_15_H_8_O_5_	Stem, Fruits	[46]
	99	Rubiadin	C_15_H_10_O_4_	Roots	[29]
	100	Rubiadin-1-methyl ether	C_16_H_12_O_4_	Fruits, Roots	[37,40]
	101	Rubiadin-3-methyl ether	C_16_H_12_O_4_	N/A	[1]
	102	Rubiadin-dimethyl ether	C_17_H_14_O_4_	N/A	
	103	Tectoquinone	C_15_H_10_O_2_	Roots	
Anthrone	104	1,8-Dihydroxy-6-methoxy-3-methyl-9-anthrone	N/A	Fruits	[47]
	105	2,4-Dimethoxy-9-anthrone	N/A	Fruits	
Carotenoids	106	*β*-Carotene	C_40_H_56_	Leaves, Fruit	[48]
Coumarins	107	Esculetin	C_9_H_6_O_4_	Fruits	[14]
	108	Fraxidin	C_11_H_10_O_5_	Barks	[38]
	109	Isofraxidin	C_11_H_10_O_5_	Barks	
	110	Isoscopoletin	C_10_H_8_O_4_	Fruits	[14]
	111	Peucedanocoumarin III	C_21_H_22_O_7_	Fruits	
	112	Pteryxin	C_21_H_22_O_7_	Fruits	
	113	Scopoletin	C_10_H_8_O_4_	Leaves, Fruit	[35]
Fatty acids	114	Caproic acid	C_6_H_12_O_2_	Fruits	[14]
	115	Caprylic acid	C_8_H_16_O_2_	Fruits	[45]
	116	Eicosanoic acid	C_20_H_40_O_2_	Fruits	[49]
	117	Hexanoic acid	N/A	Fruits	[45]
	118	Lauric acid	C_12_H_24_O_2_	Seeds	[49]
	119	Linoleic acid	C_18_H_32_O_2_	Seeds	
	120	Methyl octanoate	C_9_H_18_O_2_	Fruits	[45]
	121	Octanoic	C_8_H_16_O_2_	Fruits	
	122	Oleic acid	C_18_H_34_O_2_	Seeds	[49]
	123	Palmitoleic acid	C_16_H_30_O_2_	Seeds	
	124	Stearic acid	C_18_H_36_O_2_	Seeds	
Flavonoids	125	5,8-Dimethyl-apigenin 4′-*O*-*β*-D-galacatopyranoside	N/A	Flowers	[14]
	126	Acacetin-7-*O*-*β*-D-glucopyranoside	N/A	Fruits, Flowers	
	127	Anthocyanin	C_27_H_31_O_16_	Fruits	[15]
	128	Apigenin-5,7-dimethyl-4′-*O*-*β*-galactopyranoside	N/A	Fruits	[14]
	129	Catechin	C_15_H_14_O_6_	Fruits	[50]
	130	Epicatechin	C_15_H_11_O_6_	Fruits	[51]
	131	Kaempferol	C_15_H_10_O_6_	Leaves, Fruits	[31]
	132	Kaempferol-3-*O-α*-l-rhamnopyranosyl-(1→6)-*β*-D-glucopyranoside	N/A	Leaves, Fruits	
	133	Narcissoside	C_28_H_32_O_16_	Fruits	[30]
	134	Nicotifloroside	C_27_H_30_O_15_	Fruits	
	135	Quercetin	C_15_H_10_O_7_	Fruits	[35]
	136	Quercetin-3-*O-α*-L-rhamnopyranosyl-(1→6)-*β*-D-glucopyranoside	N/A	Leaves, Flowers	[14,52]
	137	Quercetin-3-*O*-*β*-D-glucopyranoside	C_21_H_20_O_12_	Fruits	[52]
	138	Rutin	C_27_H_30_O_16_	Fruits, Leaves	[35]
Glycosides	139	(*2E*,*4E*,*7Z*)-Deca-2,4,7-trienoate-2-*O*-*β*-D-glucopyranosyl-*β*-D-glucopyra-noside	C_22_H_34_O_13_	Fruits	[53]
	140	3-Methylbut-3-enyl 6-*O*-*β*-D-glucopyranosyl-*β*-D-glucopyranoside	C_17_H_30_O_11_	Fruits	[54]
	141	6-*O*-(*β*-D-glucopyranosyl)-1-*O*-octanoyl-*β*-D-glucopyranose	C_20_H_36_O_12_	Fruits	
	142	6-*O-(β*-D-glucopyranosyl)-1-O-hexanoyl-*β*-D-glucopyranose	C_18_H_32_O_12_	Fruits	
	143	Amyl-1-*O*-*β*-D-apio-furanosyl-1,6-*O*-*β*-D-glucopyranoside	C_16_H_30_O_10_	Fruits	[53]
Iridoids	144	6*α*-Hydroxyadoxoside	N/A	Fruits	[30]
	145	6*β*,7*β*-Epoxy-8-*epi*-splendoside	N/A	Fruits	
	146	9-*epi*-6*α*-Methoxy geniposidic acid	N/A	Fruits	[55]
	147	Asperuloside	C_18_H_22_O_11_	Fruits, Roots	[37]
	148	Asperulosidic acid	C_18_H_24_O_12_	Fruits, Roots	[35]
	149	Asperulosidic acid methyl ester	N/A	Fruits, Leaves	[52]
	150	Aucubin	C_15_H_22_O_9_	Fruits	[1]
	151	Borreriagenin	C_10_H_14_O_5_	Fruits	[30]
	152	Citrifolinin B	N/A	Leaves	[56]
	153	Citrifolinoside A	C_26_H_28_O_14_	Leaves	[57]
	154	Citrifoside	N/A	Leaves	[32]
	155	Deacetyl asperulosidic acid	C_16_H_22_O_11_	Fruits	[35]
	156	Deacetylasperuloside	N/A	Fruits, Leaves	[52]
	157	Dehydromethoxygaertneroside	N/A	Fruits	[30]
	158	Diacetylasperulosidic acid	C_16_H_22_O_11_	Roots	[37]
	159	*epi*-Dihydrocornin	N/A	Fruits	[30]
	160	Geniposidic acid	C_16_H_22_O_10_	Roots	[37]
	161	Harpagide acetate	C_17_H_26_O_11_	Roots	
	162	Isoasperulosidic acid	C_18_H_24_O_12_	Roots	
	163	Loganic acid	C_16_H_24_O_10_	Seeds	[58]
	164	Monotropein	C_16_H_22_O_11_	Roots	[37]
	165	Morindacin	C_10_H_14_O_5_	Fruits	[59]
	166	Rehmannioside A	C_21_H_32_O_15_	Roots	[37]
	167	Rhodolatouside	N/A	Seeds	
	168	Scandoside methyl ester	C_17_H_24_O_11_	Fruits	[55]
	169	Ursolic Acid	C_30_H_48_O_3_	Fruits	[60]
Lactone	170	3,4,3′,4′-Tetrahydroxy-9,7′*α*-epoxylignano-7*α*,9′-lactone	C_18_H_16_O_7_	Fruits	
Lignans	171	(-)-3,3′-Bisdemethylpinoresinol	C_18_H_18_O_6_	Fruits	
	172	(*7R*,*8R*)-3,4,9-Trihydroxy-4′,7-epoxy-8,3′-oxyneolignan-1′-al	C_16_H_14_O_6_	Fruits	
	173	(*7R*,*8R*)-3-Methoxy-1′-carboxy-4′,7-epoxy-8,3′-oxyneolignan-4,9-diol	C_17_H_16_O_7_	Fruits	
	174	Americanin A	C_18_H_16_O_6_	Fruits	[30]
	175	Episesamin 2,6-dicatechol	N/A	Fruits	[60]
	176	Isoprincepin	C_27_H_26_O_9_	Fruits	[61]
	177	Lirioresinol B	N/A	Fruits	[60]
	178	Morindolin	N/A	Fruits	[61]
	179	Pinoresinol	C_20_H_22_O_6_	Fruits	[60]
	180	*trans*-(*3*)*E*-3-(3,4-dihydroxybenzylidene)-5-(3,4-dihydroxyphenyl)-4-(hydroxymethyl) dihydrofuran-2(*3H*)-one	C_18_H_16_O_7_	Fruits	[60]
	181	*β*-Hydroxypropiovanillone	N/A	Fruits	[14]
Minerals	182	Calcium	N/A	Leaves, Fruits	[62]
	183	Cobalt	N/A	Fruits	[1]
	184	Copper	N/A	Fruits	[62]
	185	Iron	N/A	Fruits	
	186	Magnesium	N/A	Fruits	
	187	Molybdenum	N/A	Fruits	[1]
	188	Phosphor	N/A	Fruits	[62]
	189	Potassium	N/A	Fruits	[63]
	190	Selenium	N/A	Fruits	[1]
	191	Sodium	N/A	Fruits	
	192	Zinc	N/A	Fruits	
Nucleoside	193	Cytidine	C_9_H_13_N_3_O_5_	Leaves	[52]
Phenolic acid	194	Chlorogenic acid	C_16_H_18_O_9_	Fruits	[64]
	195	Gentisic acid	C_7_H_6_O_4_	Fruits	
	196	p-Hydroxybenzoic acid	C_7_H_6_O_3_	Fruits	
Phenylpropanoids	197	Butyl 3-(2,4-dihydroxy-5-methoxyphenyl) propionate	C_14_H_20_O_5_	Fruits	[65]
	198	Methyl 3-(2,4-dihydroxy-5-methoxyphenyl) propionate	C_11_H_14_O_5_	Fruits	
Saccharides	199	D-Glucose	C_6_H_12_O_6_	Fruits	[30]
	200	Methyl *β*-D-fructofuranoside	C_7_H_14_O_6_	Fruits	
	201	Methyl *α*-D-fructofuranoside	C_7_H_13_O_6_	Fruits	[1]
	202	Nonioside A	C_17_H_30_O_11_	Fruits	
	203	Nonioside B	C_26_H_46_O_17_	Fruits	
	204	Nonioside C	C_20_H_36_O_12_	Fruits	
	205	Nonioside D	C_18_H_32_O_12_	Fruits	
	206	Noniosides E	C_24_H_42_O_17_	Fruits	[66]
	207	Noniosides F	C_28_H_50_O_17_	Fruits	
	208	Noniosides G	C_34_H_60_O_18_	Fruits	
	209	Noniosides H	C_32_H_56_O_18_	Fruits	
Sterols	210	*β*-sitosterol 3-O-*β*-D-glucopyranoside	C_35_H_60_O_6_	Fruits	[30]
	211	Stigmasterol	C_29_H_48_O	Leaves	[67]
Vitamins	212	Ascorbic acid (C)	C_6_H_8_O_6_	Fruits	[14]
	213	Biotin (B7)	C_10_H_16_N_2_O_3_S	Fruits	[1]
	214	Cobalamin (B12)	C_63_H_88_CoN_14_O_14_P	Fruits	
	215	Folic Acid (B9)	C_19_H_19_N_7_O_6_	Fruits	
	216	Niacin (B3)	C_6_H_5_NO_2_	Fruits	
	217	Pantothenic Acid (B5)	C_9_H_17_NO_5_	Fruits	
	218	Pyridoxine (B6)	C_8_H_11_NO_3_	Fruits	
	219	Riboflavin (B2)	C_17_H_20_N_4_O_6_	Fruits	
	220	Thiamine (B1)	C_12_H_17_N_4_OS	Fruits	
	221	Tocopherol (E)	C_29_H_50_O_2_	Fruits	
	222	Vitamin K	C_31_H_46_O_2_	Fruits	
Others	223	2,6-Di-*O*-(*β*-D-glucopyranosyl)-1-*O*-octanoy-1-*β*-D-glucopyranose	N/A	N/A	[14]
	224	4-*O*-*β*-D-glucopyranosyl-(1→4)-*α*-L-rhamnopyranoside	N/A	Flowers	
	225	Morinaphthalene	N/A	Fruits	[47]
	226	Morinaphthalenone	N/A	Fruits	
	227	Morindafurone	N/A	Fruits	
	228	Morinthone	C_31_H_44_O_3_	Fruits	[14]
	229	*β*-D-glucopyranose-penta-acetate	N/A	Fruits	

Notes: N/A, data not available.

**Table 2:** Anthraquinones isolated from *M. citrifolia*.

Anthraquinones	R^1^	R^2^	R^3^	R^4^	R^5^	R^6^	R^7^	R^8^
1,2-Dihydroxy-3-methoxy-anthraquinone	OH	OH	OCH_3_	H	H	H	H	H
1,3,6-Trihydroxy-2-methylanthraquinone	OH	CH_3_	OH	H	H	OH	H	H
1,3-Dihydroxy-2-methoxy-anthraquinone	OH	OCH_3_	OH	H	H	H	H	H
1,3-Dihydroxy-5-methoxy-2,6-bismethoxymethyl-9,10-anthraquinone	OH	CH_2_OCH_3_	OH	H	OCH_3_	CH_2_OCH_3_	H	H
1,3-Dihydroxy-5-methoxy-6-methoxymethyl-2-methyl-9,10-anthraquinone	OH	CH_3_	OH	H	OCH_3_	CH_2_OCH_3_	H	H
1,3-Dimethoxy-2-methoxymethylanthraquinone	OCH_3_	CH_2_OCH_3_	OCH_3_	H	H	H	H	H
1,3-Dimethoxyanthraquinone	OCH_3_	H	OCH_3_	H	H	H	H	H
1,4-Dimethoxyl-2-hydroxyanthraquinone	OCH_3_	OH	H	OCH_3_	H	H	H	H
1,5,15-Trimethylmorindol	OCH_3_	CH_2_OCH_3_	H	H	OCH_3_	OH	H	H
1,5,7-Trihydroxy-6-methoxy-2-methoxymethylanthraquinone	OH	OCH_3_	OH	H	OH	CH_2_OCH_3_	H	H
1,8-Dihydroxy-2-hydroxymethyl-5-methoxyanthraquinone	OH	CH_2_OH	H	H	OCH_3_	H	H	OH
1,8-Dihydroxy-2-methyl-3,7-dimethoxyanthraquinone	OH	CH_3_	OCH_3_	H	H	H	OCH_3_	OH
1-Hydroxy-2-methyl-9,10-anthraquinone	OH	CH_3_	H	H	H	H	H	H
1-Hydroxy-2-primeverosyloxymethyl-anthraquinone-3-olate	OH	CH_2_O-prime	O	H	H	H	H	H
1-Hydroxy-5,6-dimethoxy-2-methyl-7-primeverosyloxyanthraquinone	OH	CH_3_	H	H	OCH_3_	OCH_3_	O-prime	H
1-Hydroxy-5-methoxyanthraquinone	OCH_3_	H	H	H	OH	H	H	H
1-Methoxy-2-primeverosyloxymethyl-anthraquinone-3-olate	OCH_3_	CH_2_O-prime	O	H	H	H	H	H
1-Methoxy-3-hydroxyanthraquinone	OCH_3_	H	OH	H	H	H	H	H
1-Methyl-3-hydroxy-anthraquinone	CH_3_	H	OH	H	H	H	H	H
1-*O*-gentiobiose-2-methylol-anthraquinone	O-gent	CH_2_OH	H	H	H	H	H	H
1-*O*-gentiobiose-3-hydroxy-2-methyl-anthraquinone	O-gent	CH_3_	OH	H	H	H	H	H
1-*O*-gentiobiose-8-methoxy-aloeemodin	O-gent	H	CH_2_OH	H	H	H	H	OCH_3_
1-*O*-gentiobiose-emodin	O-gent	H	CH_3_	H	H	OH	H	OH
1-*O*-primeverose-2-methyl-3,6,8-trihydroxy-anthraquinone	O-prime	CH_3_	OH	H	H	OH	H	OH
1-*O*-primeverose-2-methylol-3-hydroxy-8-methoxy-anthraquinone	O-prime	CH_2_OH	OH	H	H	H	H	OCH_3_
1-*O*-primeverose-2-methylol-anthraquinone	O-prime	CH_2_OH	H	H	H	H	H	H
1-*O*-primeverose-3,8-dihydroxy-2-methyl-anthraquinone	O-prime	CH_3_	OH	H	H	H	H	OH
1-*O*-primeverose-3,8-dimethoxy-2-methyl-anthraquinone	O-prime	CH_3_	OH	H	H	H	H	OCH_3_
1-*O*-primeverose-3-methoxy-8-hydroxy-2-methylol-anthraquinone	O-prime	CH_2_OH	OCH_3_	H	H	H	H	OH
1-*O*-primeverose-8-hydroxy-ibericin	O-prime	CH_2_OCH_2_CH_3_	OH	H	H	H	H	OH
1-*O*-primeverose-aloeemodin	O-prime	H	CH_2_OH	H	H	H	H	OH
1-*O*-primeverose-emodin	O-prime	H	CH_3_	H	H	OH	H	OH
1-*O*-primeverose-rubiadin	O-prime	CH_3_	OH	H	H	H	H	H
1-*O*-*β*-D-glycopyranosyl-8-methoxy-emodin	O-glu	H	CH_2_OH	H	H	OH	H	OCH_3_
1-*O*-*β*-D-glycopyranosyl-emodin	O-glu	H	CH_3_	H	H	OH	H	OH
1-*O*-*β*-D-glycopyranosylrubiadin-3-methyl ether	O-glu	CH_3_	OCH_3_	H	H	H	H	H
2-Ethoxy-1-hydroxyanthraquinone	OH	OCH_2_CH_3_	H	H	H	H	H	H
2-Formyl-1-hydroxyanthraquinone	OH	CHO	H	H	H	H	H	H
2-Formylanthraquinone	H	CHO	H	H	H	H	H	H
2-Methoxy-1,3,6-trihydroxyanthraquinone	OH	OCH_3_	OH	H	H	OH	H	H
2-Methoxy-3-methyl-anthraquinone	H	OCH_3_	CH_3_	H	H	H	H	H
3-*O*-gentiobiose-1-hydroxy-2-methyl-anthraquinone	OH	CH_3_	O-gent	H	H	H	H	H
3-*O*-primeverose-1,6,8-trihydroxy-2-methyl-anthraquinone	OH	CH_3_	O-prime	H	H	OH	H	OH
5,15-Di-*O*-methylmorindol	OH	CH_2_OCH_3_	H	H	OCH_3_	OH	H	H
6-Hydroxy-anthragallol-1,3-dimethylether	OCH_3_	OH	OCH_3_	H	H	OH	H	H
8-*O*-gentiobiose-emodin	OH	H	CH_3_	H	H	OH	H	O-gent
Alizarin	OH	OH	H	H	H	H	H	H
Alizarin-1-methyl ether	OCH_3_	OH	H	H	H	H	H	H
Aloe-emodin	OH	H	CH_2_OH	H	H	H	H	OH
Anthragallol-2-methyl ether	OH	OCH_3_	OH	H	H	H	H	H
Damnacanthal	OCH_3_	CHO	OH	H	H	H	H	H
Emodin	OH	H	OH	H	H	CH_3_	H	OH
Emodin 1-*O*-*β*-D-glycopyranosyl	OH	H	CH_3_	H	H	OH	H	O-glu
Fridamycin E	OH	CH_2_COH(CH_3_)CH_2_COOH	H	H	OH	H	H	H
Ibericin	OH	CH_2_OCH_2_CH_3_	OH	H	H	H	H	H
Lucidin	OH	CH_2_OH	OH	H	H	H	H	H
Lucidin 3-methyl ether	OH	CH_2_OH	OCH_3_	H	H	H	H	H
Morindicininone	OCH_3_	H	OCH_3_	CH_2_OH	H	H	H	H
Morindicinone	OCH_3_	OH	H	H	H	H	CH_2_OCH_3_	OCH_3_
Morindone	OH	OH	H	H	OH	CH_3_	H	H
Morindone-5-methylether	OH	CH_3_	H	H	OCH_3_	OH	H	H
Morindone-6-methyl-ether	OH	CH_3_	H	H	OH	OCH_3_	H	H
Morindone-6-*O*-*β*-D-primeveroside	OH	CH_3_	H	H	OH	O-prime	H	H
Nordamnacanthal	OH	CHO	OH	H	H	H	H	H
Rubiadin	OH	CH_3_	OH	H	H	H	H	H
Rubiadin-1-methyl ether	OCH_3_	CH_3_	OH	H	H	H	H	H
Rubiadin-3-methyl ether	OH	CH_3_	OCH_3_	H	H	H	H	H
Rubiadin-dimethyl ether	OCH_3_	CH_3_	OCH_3_	H	H	H	H	H
Tectoquinone	H	CH_3_	H	H	H	H	H	H

**Table 3:** Summary of antioxidant activity studies of *M. citrifolia*.

No.	Parts	Animal/Cell	Model	Application Part or Compounds	Dose	Pharmacological Activity	Reference
1	Leaves	N/A	In vitro: DPPH, ABTS	*M. citrifolia* leaf extract silver nanoparticles	17.70, 13.37 μg/mL	Scavenging radical	[98]
2	Fruits	N/A	In vitro: DPPH, ABTS	Polysaccharide	0.2–1 mg/mL	Scavenging radical	[100]
3	Fruits	N/A	In vitro: DPPH	PTP	3.91–2000 µg/mL	Scavenging radical	[99]
4	Fruits	C57BL/6 mice	High-fat diet-induced fatty liver	Phenolic extract	100, 200 mg/kg	Elevating MDA levels and decreasing GSH levels and CAT activity in mice with NAFLD	[97]
5	Fruits	C57BLKS mice, HepG2 cells	In vitro: 50 mM high glucose established insulin-resistant In vivo: 10 mg/kg/d rosiglitazone (ROSI)-induced oxidative stress model	Fermented noni juice (FNJ)	6.5, 13 mL/kg	Activation of the Nrf2/ARE (Antioxidant Response Element) signaling pathway and modulation of intestinal flora in db/db mice and IR-HepG2 cells	[101]
6	Fruits	N/A	In vitro: hydroxyl radical scavenging capacity, superoxide anion scavenging capacity and anti-lipid peroxidation capacity	Polysaccharide	0.2–3.2 g/L	Scavenging radical	[20]
7	Leaves, Fruits	N/A	In vitro: DPPH	Methanol extract	300 µg/mL	Scavenging radical	[104]
8	Leaves	N/A	In vitro: DPPH, ABTS	Lignin	175.34 μg/mL	Scavenging radical	[103]
9	Leaves	The freshwater catfish, *P. sutchi*	Freshwater catfish fed 5.75 mg/L cadmium	Methanol extract	200 mg/kg	Reducing oxidative stress in a time-dependent manner	[105]
10	Fruits	N/A	Total antioxidant capacity test	*M. citrifolia* fruit ethanol extracts	0.823–2.106 U/mL	Antioxidant activity	[106]
11	Leaves	N/A	Phospho molybdenum-based method	Flavonoid-rich *M. citrifolia* methanol extracts	350 μg/mL	Intracellular membrane damage, cell cycle blockade	[107]
12	Fruits	C57BL/6J mice	High-fat diet-induced oxidative stress and disorders of lipid metabolism	Fruit wine	10, 20, 40 mL/kg	Reduction of hepatic ROS and MDA levels and increasing serum and hepatic antioxidant enzyme activities	[78]
13	Fruits	Sprague-Dawley (SD) Rat	High-fat diet-induced oxidative stress and disorders of lipid metabolism	Polysaccharide	100 mg/kg	Modulation of intestinal flora and SCFA production reduction of colonic barrier permeability and metabolic endotoxemia, thereby attenuating hepatic oxidative stress and inflammation in High-Fat Diet (HFD) rats	[79]
14	Fruits	N/A	In vitro: DPPH, ABTS	Polysaccharide	0.2–5 mg/mL	Scavenging radical	[80]
15	Fruits	Kunming mice	High-fat diet-induced oxidative stress and disorders of lipid metabolism	Noni fruit water extract (NFW), Noni fruit polysaccharide (NFP)	50, 100, 200 mg/kg	Increasing the hepatic nuclear factor erythroid-2-related factor level	[81]
16	Leaves	SD rat	Obesity and hepatic oxidative stress induced by thermoxidized palm oil diet	Ethanol extract	500, 1000 mg/kg	Elevating liver antioxidant enzymes	[102]

Notes: N/A, data not available.

**Table 6:** Summary of studies on hypoglycemic activity of *M. citrifolia*.

No.	Parts	Animal/Cell	Model	Application Part or Compounds	Dose	Pharmacological Activity	Reference
1	Fruits	Mice	Streptozotocin-induced diabetes	Ursolic acid, sterol compound	N/A	Inhibiting glucose	[133]
2	Seeds	N/A	N/A	Compounds isolated by ethyl acetate and petroleum ether extraction of noni seeds	160, 133, 120 μmol/L	*α*-Glucosidase inhibitory activity	[134]
3	Fruits	SD rat	Diabetes conditions were induced by streptozotocin	Plant extracts made up of noni (*M. citrifolia*), pineapple (*Ananas comosus*), and mango (*Mangifera indica*)	4.04 ± 0.03 mg/mL 3.42 ± 0.02 mg/mL	Inhibition of *α*-glucosidase and *α*-amylase	[135]
4	Fruits	Swiss mice	High-sugar, high-fat diet (21.20% carbohydrate; 18.86% protein; 4% soybean oil, 31% lard, and 20% fructose)	Fruit water extract	500 mg/kg	Up-regulation of peroxisome proliferator–activated receptor alpha (PPAR-α) expression in adipose tissue and down-regulation of peroxisome proliferator–activated receptor gamma (PPAR-γ), PPAR-α, sterol regulatory element binding protein-1c (SREBP-1c), and fetuin-A expression in liver	[136]

Notes: N/A, data not available.

**Table 7:** Summary of studies on the antimicrobial activity of *M. citrifolia*.

No.	Parts	Animal/Cell/Microbiology	Method	Application Part or Compounds	Inhibition Zone	Pharmacological Activity	Reference
1	Roots	*B. cereus*, *S. aureus*, *S. epidermidis*, *E. coli*, *M. tuberculosis*, and *P. aeruginosa*	Disk diffusion method	Dichloromethane root extract (10 μL) hexane root extract (10 μL) ethyl acetate root extract (10 μL)	*E. coli* (5.5 mm) *S. aureus* (4.0 mm) *B. cereus* (5.0 mm) *P. aeruginosa* (5.5 mm)	Inhibitory of *E. coli*, *S. aureus*, *B. cereus*, and *P. aeruginosa*	[3]
2	*M. citrifolia* extract	*S. aureus*, *P. aeruginosa*, and *C. albicans*	Minimum inhibitory concentration (MIC)	*M. citrifolia* extract (6.25%), zinc oxide	*S. aureus* (16.33 mm) *P. aeruginosa* (28.5 mm) *C. albicans* (10 mm)	Strong inhibitory effect on *S. aureus*, *P. aeruginosa*, and *C. albicans*	[139]
3	Fruits	*Fusobacterium*, *Candida albicans*, and *Prevotella*	Antimicrobial susceptibility testing	Noni fruit juice	*Fusobacterium* (12.8 mm) *Candida albicans* (11.6 mm) *Prevotella* (12.4 mm)	Antibacterial activity	[140]

## Abbreviations

The following abbreviations are used in this manuscript:

A-549	Human non-small cell lung cancer cells
ABTS	2,2′-azino-bis(3-ethylbenzothiazoline-6-sulfonicacid)
AP-1	Activator Protein-1
ARE	Antioxidant Response Element
CAT	Catalase
CNKI	China National Knowledge Infrastructure
DAA	Deacetylasperulosidic Acid
DNCB	2,6-Dinitro-1-chlorobenzene
DPPH	2,2-Diphenyl-1-picrylhydrazyl
EMA	European Medicine Agency
EOL-1	Eosinophilic Leukemia-1
FDA	Food and Drug Administration
FNJ	Fermented Noni Juice
GPx	Glutathione Peroxidase
GSH	Glutathione
HaCaT	Human Adult Cutaneous T-Cell Lymphoma
HCT116	HCT116 human colon cancer cell line
HFD	High-Fat Diet
HL-60	HL-60 human promyelocytic leukemia cell line
HMC-1	Human Mast Cell-1
HPLC	High-Performance Liquid Chromatography
HRFAB	High-Resolution Fast Atom Bombardment
HT29	HT29 human colon cancer cell line
IBD	Inflammatory Bowel Disease
IFN-γ	Interferon-γ
IL-10	Interleukin-10
IL-12	Interleukin-12
IL-1β	Interleukin-1 beta
IL-6	Interleukin-6
IL-8	Interleukin-8
iNOS	Inducible Nitric Oxide Synthase
Keap1	Kelch-like ECH-associated protein 1
KRAS	Kirsten Rat Sarcoma Viral Oncogene Homolog
LNCaP	Human prostate cancer
LPS	Lipopolysaccharide
LS174T	LS174T human colon cancer cell line
*M. citrifolia*	*Morinda citrifolia* L.
MAE	Microwave-Assisted Extraction
MAPK	Mitogen-Activated Protein Kinase
MCEO-CHs NPs	Nanoparticles of noni fruit essential oil loaded with chitosan
MCF-7	Michigan Cancer Foundation-7
McLTP_1_	*M. citrifolia* Lipid Transfer Protein
MDA	Malondialdehyde
MDM2-p53	Murine Double Minute 2-p53 protein
MIC	Minimum Inhibitory Concentration
MMP-1	Matrix Metalloproteinase-1
MPLC	Medium-Pressure Liquid Chromatography
MS	Mass Spectrometry
NAFLD	Non-Alcoholic Fatty Liver Disease
NLRP3	NLR family pyrin domain-containing protein 3
NFP	Noni Fruit Polysaccharide
NFW	Noni Fruit Water extract
NF-κB	Nuclear Factor kappa-B
NK cell	Natural Killer cell
NMPA	National Medical Products Administration
NMR	Nuclear Magnetic Resonance
NO	Nitric Oxide
NOS	Nitric Oxide Synthase
Nrf2	Nuclear factor erythroid 2-related factor 2
OTC	Over The Counter
PPAR-α	Peroxisome Proliferator–Activated Receptor alpha
PPAR-γ	Peroxisome Proliferator–Activated Receptor gamma
PRISMA	Preferred Reporting Items for Systematic Reviews and Meta-Analysis
PTP	Phikud Tri-Phon
ROS	Reactive Oxygen Species
ROSI	Rosiglitazone
SCFAs	Short-Chain Fatty Acids
SD	Sprague-Dawley
SKG	Saccharomyces cerevisiae Kinase Gene
SMMC-7721	Shanghai Medical College of Fudan University Hepatocellular Carcinoma-7721
SOD	Superoxide Dismutase
SREBP-1c	Sterol Regulatory Element Binding Protein-1c
SW480	SW480 human colon cancer cell line
Th1	T helper cell 1
Th17	T helper cell 17
Th2	T helper cell 2
Th22	T helper cell 22
THP-1	Tohoku Hospital Pediatrics-1
TLC	Thin-Layer Chromatography
TNF-α	Tumor Necrosis Factor-α
UAE	Ultrasound-Assisted Extraction
UV	Ultraviolet
WEHI-3B	Walter and Eliza Hall Institute-3B
